# Long-term clinical outcome in patients with acute coronary syndrome and dysglycaemia

**DOI:** 10.1186/s12933-015-0283-3

**Published:** 2015-09-17

**Authors:** Jeanette Kuhl, Gun Jörneskog, Malin Wemminger, Mattias Bengtsson, Pia Lundman, Majid Kalani

**Affiliations:** Division of Cardiovascular Medicine, Department of Clinical Sciences, Karolinska Institutet, Danderyd Hospital, Stockholm, Sweden; Department of Medicine, Karolinska Institutet, Karolinska University Hospital, 182 88 Stockholm, Sweden; Division of Medicine, Department of Clinical Sciences, Karolinska Institutet, Danderyd Hospital, Stockholm, Sweden

**Keywords:** Cardiovascular disease, Diabetes mellitus, Impaired glucose tolerance, Prognosis

## Abstract

**Background:**

Diabetes and impaired glucose tolerance (IGT) are major risk factors for atherosclerosis including coronary artery disease (CAD). The present study’s aim was to investigate the importance of glucose tolerance for long-term clinical outcome in patients with acute coronary syndrome (ACS).

**Methods:**

A total 1062 consecutive patients, 781 men and 281 women, aged 32–80 years, admitted to the coronary care unit at Danderyd University Hospital, Stockholm, for ACS from 2006 to 2008 were included. At discharge, the patients were categorized according to an oral glucose tolerance test (OGTT) as having normal glucose tolerance (NGT), n = 295 (28 %); impaired fasting glucose (IFG) and IGT, n = 299 (28 %); diabetes discovered by OGTT, n = 156 (15 %); or known diabetes at admission, n = 312 (29 %). Mortality and reinfarction rates were studied during a mean follow-up time of 4.0 (±0.8) years. Clinical outcome data were obtained from the Swedish Coronary Angiography and Angioplasty Registry and the Swedish National Registry.

**Results:**

There was significantly higher (p < 0.001) mortality within, 30 days, 1 and 3 years in patients with known diabetes as compared to the other groups. During the follow-up, 86 patients (28 %) with known diabetes had reinfarction as compared to 36 patients (12 %) with NGT and 79 patients (17 %) with dysglycaemia (IFG, IGT and diabetes) discovered by OGTT.

**Conclusion:**

A majority (72 % in this study) of patients admitted for ACS have disturbed glucose metabolism, including diabetes, with high prevalence of previously undiagnosed dysglycaemia. Both patients with known diabetes and dysglycaemia discovered by OGTT show a high risk for poor clinical prognosis.

## Background

There is a global increase in the prevalence of type 2 diabetes mellitus (T2DM) and impaired glucose tolerance (IGT), which are major risk factors for atherosclerosis including acute coronary syndrome (ACS) caused by coronary artery disease (CAD) [1, 2]. T2DM and IGT have been shown to occur among as many as two-thirds of patients with ACS and previously unknown diabetes [3, 4]. A long-term follow-up of these patients has demonstrated that these newly detected glucose disturbances also affect future prognosis [4].

The Euro Heart Survey shows that a majority of patients diagnosed with CAD including ACS have abnormal glucose regulation [5], and two earlier studies that included previously healthy individuals showed that IGT leads to an increased risk of cardiovascular disease (CVD) and death [6, 7]. There are several factors contributing to why patients with T2DM and IGT are particularly prone to develop ACS. Among these are vascular endothelial dysfunction, disturbed platelet function, decreased fibrinolytic capacity, high levels of advanced glycation end products (AGEs), dyslipidemia, hypertension and reduced insulin sensitivity [8–12].

T2DM develops slowly through stages of early impairments of glucose metabolism. A majority of subjects who develop T2DM demonstrate IGT approximately 5 years prior to diagnosis [13]. With regard to ACS, the research to date has tended to focus on diabetes rather than IGT. Thus, the impact of IGT on ACS, and cardiovascular morbidity and mortality in general, is yet to be revealed. A large study of patients with CAD showed that diabetes, whether it is previously known or newly detected, is an independent risk factor for mortality and myocardial infarction during a 1-year follow-up period [14]. Impaired glucose regulation, including IGT and impaired fasting glucose (IFG), was not an independent predictor of adverse outcomes in these patients [14]. However, over a longer period of time, glucose levels far below the threshold for diabetes have been shown to markedly increase the risk of cardiovascular events [15].

The primary aim of the present study was to investigate the importance of dysglycaemia detected by an oral glucose tolerance test (OGTT) for long-term mortality and cardiovascular events in patients with ACS.

## Methods

### Patients

Included in the study were 1062 consecutive patients, 281 women and 781 men, aged 32–80 years, admitted to the coronary care unit at Danderyd Hospital for myocardial infarction or unstable angina between January 19, 2006 and December 29, 2008. All patients, except patients with known type 1 or type 2 diabetes, underwent a standardised 75-g OGTT according to WHO criteria [16] at 4–5 days after admission. According to the American Diabetes Association document from 1997 [17], the criteria for IFG were a fasting plasma glucose level of ≥6.1 to <7.0 mmol/l and a 2-h plasma glucose level at OGTT <7.8 mmol/l; and for IGT, a fasting plasma glucose level <6.1 mmol/l and a 2-h plasma glucose level at OGTT of ≥7.8 to <11.1 mmol/l. T2DM was defined as having a fasting plasma glucose value ≥7.0 mmol/l, and/or a 2-h plasma glucose value at OGTT ≥11.1 mmol/l.

Myocardial infarction and unstable angina pectoris were defined according to the criteria recommended by the Joint European Society of Cardiology and the American College of Cardiology Committee [18].

### Protocol

Data were collected in two steps:

1. Baseline data and OGTT results were collected at the time of admission from 2006 to 2008.
2. Retrospective review of the Swedish Web system for Enhancement and Development of Evidence-based Care in Heart Disease Evaluated According to Recommended Therapies Registry (SWEDEHEART) and the Swedish Coronary Angiography and Angioplasty Registry (SCAAR) was performed in 2012.

### Statistical analysis

Data are presented as mean ± standard deviation (SD) or in percentage when categorical values. The Kruskal–Wallis test followed by Mann–Whitney’s U test (Table 1) or Chi square test (Tables 1, 2, 3, 4) was performed to analyse differences in continuous variables between groups. Data shows absolute risks. Statistical significance was set at p < 0.05. Data processing was performed using IBM SPSS, version 22.

**Table 1:** Basic characteristics in 1062 patients at time of admission for acute coronary syndrome

	NGT	IFG/IGT	Diabetes^A^	Diabetes^B^	P
No.	295	299	156	312	
Female/male (n)	76/219	77/222	40/116	88/224	NS
Age (years)	61.4 (9.81)^e,h,l^	63.9 (10.40)^b,l^	64.3 (8.77)^b,k^	66.9 (8.99)^c,f,h^	<0.001
BMI (kg/m^2^)	25.9 (3.96)^e,i,l^	27.1 (3.92)^b,k^	27.8 (3.81)^c^	28.5 (5.00)^c,e^	<0.001
Systolic blood pressure^m^ (mm/Hg)	143.9 (27.84)	148.7 (28.87)	147.6 (29.69)	144.8 (29.77)	NS
Diastolic blood pressure^m^ (mm/Hg)	82.9 (14.26)	85.4 (15.49)^l^	83.6 (13.90)	80.2 (15.34)^f^	<0.001
S-Creatinine (µmol/L)	84.0 (19.95)^l^	86.7 (33.40)^l^	81.9 (17.44)^l^	111.6 (89.31)^c,f,i^	<0.001
Previous (%)					
Acute myocardial infarction	14.7^l^	19.6^l^	12.9^l^	52.7^c,f,h^	<0.001^Ω^
Hypertension	16.7^f,l^	25.7^c,i,l^	16.9^f,l^	40.7^c,f,i^	<0.001^Ω^
CABG	14.6^l^	17.7^l^	14.6^l^	53.1^c,f,h^	<0.001^Ω^
PCI	21.1^l^	17.9^l^	15.4^l^	45.5^c,f,h^	<0.001^Ω^
Smokers	26.9^g^	26.7^g^	15.8^a,d,j^	30.6^g^	<0.05^Ω^

Values are means (SD). Presented values are unadjusted

*CABG* coronary artery bypass graft,* PCI* percutaneous coronary intervention

vs NGT, ^a^p < 0.05, ^b^ p < 0.01, ^c^ p < 0.001; vs IFG/IGT, ^d^ p < 0.05, ^e^ p < 0.01, ^f^ p < 0.001; vs Diabetes OGTT, ^g^ p < 0.05, ^h^ p < 0.01, ^i^ p < 0.001; vs known diabetes, ^j^ p < 0.05, ^k^ p < 0.01, ^l^ p < 0.001

^m^Significance testing after adjustment for age, BMI, and sex, for the variables systolic blood pressure and diastolic blood pressure

^Ω^Chi square test

^A^Patients with diabetes discovered by OGTT

^B^Patients with known diabetes at admission

**Table 2:** In-hospital treatment and events in 1062 patients with acute coronary syndrome

				NGT	IFG/IGT	Diabetes^A^	Diabetes^B^	P
No.				295	299	156	312	
Female/male (n)				76/219	77/222	40/116	88/224	NS
Coronary angiography %				92.5 (273)^l^	94.0 (281)^l^	92.3 (144)^l^	73.7 (230)^c,f,i^	<0.001
Coronary angio-graphy in 928 patients								
PCI % (of 928)				70.7 (193)^l^	66.5 (187)^l^	69.4 (100)^l^	57.4 (132)^c,f,i^	<0.05
PCI in 612 patients								
Stent % (of 612)				87.0 (168)	87.7 (164)	87.0 (87)	89.4 (118)	NS
Stent in 537 patients								
BMS %				81.5 (137)^l^	82.9 ^l^ (136)	81.6 (71)^l^	60.2 (71)^c,f,i^	<0.001
DES %				18.5 (31)^l^	17.1^l^ (28)	18.4 (16)^l^	39.8 (47)^c,f,i^	<0.001
CABG %				13.9 (41)	16.7 (50)	16.7 (26)	17.9 (56)	NS
LVEF evaluation %				83.4 (246)^l^	83.3 (249)^l^	87.2 (136)^l^	76.0 (237)^c,f,i^	<0.001
Normal LVEF (>50 %)				74.4 (183)^l^	72.3 (180)^l^	67.6 (92)^l^	50.6 (120)^c,f,i^	<0.001
Reduced LVEF (<50 %)				25.6 (63)^l^	27.7 (69)^l^	32.4 (44)^l^	49.4 (117)^c,f,i^	<0.001

Parenthesis show number of individuals

*PCI* percutaneous coronary intervention, *BMS* bare metal stents, *DES* drug-eluting stents, *CABG* coronary artery bypass graft, *LVEF* left ventricular ejection fraction

vs NGT, ^a^p < 0.05, ^b^ p < 0.01, ^c^ p < 0.001; vs IFG/IGT, ^d^ p < 0.05, ^e ^p < 0.01, ^f ^p < 0.001; vs Diabetes OGTT, ^g^ p < 0.05, ^h ^p < 0.01, ^i^ p < 0.001; vs known diabetes, ^j^ p < 0.05, ^k ^p < 0.01, ^l ^p < 0.001

^A^Patients with diabetes discovered by OGTT

^B^Patients with known diabetes at admission

**Table 3:** Medical treatment at hospital discharge in 1062 patients with acute coronary syndrome

	NGT	IFG/IGT	Diabetes^A^	Diabetes^B^	P
No.	295	299	156	312	
Female/male (n)	76/219	77/222	40/116	88/224	NS
Treatment at discharge (%)					
Aspirin	97.8^l^	97.5^l^	96.7^l^	89.4^c,f,i^	<0.001
Clopidogrel	80.6^l^	81.1^l^	80.0^l^	66.7^c,f,i^	<0.001
Beta-blockers	94.7^l^	96.1^l^	97.3^l^	95.0^c,f,i^	NS
Calcium antagonists	9.2^l^	14.6^l^	9.3^l^	24.6^c,f,i^	<0.001
ACE inhibitors	51.2^f,i,l^	62.6^c^	70.7^c^	63.8^c^	<0.001
Angiotensin II receptor blockers	7.8^f,i,l^	12.5^c,l^	12.0^c,l^	21.9^c,f,i^	<0.001
Diuretics	8.9^f,i,l^	16.8^l^	16.7^l^	42.5^c,f,i^	<0.001
Statins	94.7^l^	92.9^l^	95.3^l^	88.0^c,f,i^	<0.01
Oral antidiabetic medication	0^i,l^	0^i,l^	7.3^c,f,l^	56.1^c,f,i^	<0.001
Insulin	0^l^	0^l^	0^l^	52.7^c,f,i^	<0.001
Oral antidiabetic medication and Insulin	0^l^	0^l^	0^l^	26.3^c,f,i^	<0.001

vs NGT, ^a^p < 0.05, ^b ^p < 0.01, ^c^ p < 0.001; vs IFG/IGT, ^d ^p < 0.05, ^e ^p < 0.01, ^f ^p < 0.001; vs Diabetes OGTT, ^g^ p < 0.05, ^h^ p < 0.01, ^i^ p < 0.001; vs known diabetes, ^j ^p < 0.05, ^k^ p < 0.01, ^l^ p < 0.001

^A^Patients with diabetes discovered by OGTT

^B^Patients with known diabetes at admission

**Table 4:** Patient mortality and reinfarction (281 women and 781 men)

	NGT	IFG/IGT and diabetes^A^	Diabetes^B^	P
No.	295	455	312	
Death within 30 days, n (%)	0^c^	1 (0.2)^c^	15 (4.8)^a,b^	<0.001
Death within 1 year, n (%)	0^c^	9 (2.0)^c^	39 (12.5)^a,b^	<0.001
Death within 3 years, n (%)	9 (3.1)^c^	23 (5.1)^c^	77 (24.7)^a,b^	<0.001
Reinfarction, n (%)	36 (12.2)^c^	79 (17.4)^c^	86 (27.6)^a,b^	<0.001

vs NGT ^a^p < 0.001; vs IFG/IGT and diabetes OGTT ^b ^p < 0.001; vs known diabetes, ^c^ p < 0.001

^A^Patients with diabetes discovered by OGTT

^B^Patients with known diabetes at admission

### Ethical considerations

All participants received written and oral information regarding the study and gave their written informed consent. The study protocol was approved by the local Ethics Committee at Karolinska Institutet.

## Results

### Glucose tolerance

Table 1 shows the distribution of women and men with ACS in all glucose tolerance groups. In total, 76 (27.0 %) women and 219 (28.0 %) men had NGT, and the corresponding figures for IFG/IGT were 77 (27.4 %) women and 222 (28.4 %) men; for patients with diabetes discovered by OGTT, numbers were 40 (14.2 %) women and 116 (14.9 %) men. Finally, 88 (31.3 %) women and 224 (28.7 %) men had known diabetes at admission to the hospital. Among patients with diabetes, 31 (9.9 %) had type 1 diabetes (data not shown).

### Basic characteristics and ACS diagnosis

Table 1 shows that individuals with disturbances of glucose tolerance were older than those with NGT (p < 0.001), and that patients with known diabetes were older than individuals with IFG/IGT as well as patients with diabetes discovered by OGTT (p < 0.01). There was a significant increase in BMI in individuals with disturbances of glucose tolerance compared to subjects with NGT (p < 0.01), and patients with known diabetes had significantly higher BMI than individuals with IFG/IGT (p < 0.01). Furthermore, diastolic blood pressure was lower in patients with known diabetes compared to individuals with IFG/IGT (p < 0.001), while there were no significant differences in systolic blood pressure levels between the four groups. At the time of admission, patients with known diabetes had higher serum creatinine levels (p < 0.001) as well as higher prevalence of previous acute myocardial infarction (AMI), coronary artery bypass graft (CABG) and percutaneous coronary intervention (PCI) (p < 0.01) compared to the three other groups. Furthermore, the prevalence of smoking was less (p < 0.05) among patients with diabetes discovered by OGTT as compared to the other groups. Table 1 also shows that the prevalence of previous hypertension was higher in patients with known diabetes compared to the other three groups (p < 0.001), and higher in individuals with IFG/IGT compared to NGT and patients with diabetes discovered by OGTT (p < 0.001).

Among individuals with NGT, 14.6 % were diagnosed with unstable angina pectoris and 85.4 % AMI, and among individuals with IFG/IGT, 11.7 % were diagnosed with unstable angina pectoris and 88.3 % AMI. The corresponding numbers among patients with diabetes discovered by OGTT were 15.4 and 84.6 %, respectively. Among patients with known diabetes at admission to the hospital, 16.0 % were diagnosed with unstable angina pectoris and 84.0 % had AMI.

### In-hospital treatment and events

Table 2 shows that patients with known diabetes less frequently underwent coronary angiography and PCI, and were more often treated with drug-eluting stents (DES) instead of bare metal stents (BMS). In total, 928 of 1062 patients underwent coronary angiography; 612 of 928 patients underwent PCI, and 537 of 612 patients were treated with stents, of which, 415 of 537 got DES, and 122 of 537 got BMS. Furthermore, the evaluation of the left ventricular ejection fraction (LVEF) was performed less in patients with known diabetes as compared to the other three groups. The prevalence of reduced ejection fraction was significantly higher in patients with known diabetes as compared to the other groups (p < 0.001).

Medical treatment at hospital discharge is shown in Table 3. Aspirin, clopidogrel and statins were used less frequently in patients with known diabetes compared to individuals with NGT, IFG/IGT and patients with diabetes discovered by OGTT. Calcium antagonists, angiotensin II receptor blockers and diuretics were more frequently used among patients with known diabetes compared to the other three groups. Finally, there was no significant difference in the use of beta-blockers, while ACE inhibitors were less frequently used among individuals with NGT. Among patients with diabetes discovered by OGTT, 7.3 % were started on oral antidiabetic medication at the time of discharge.

### Mortality and reinfarction

**Fig. 1:**
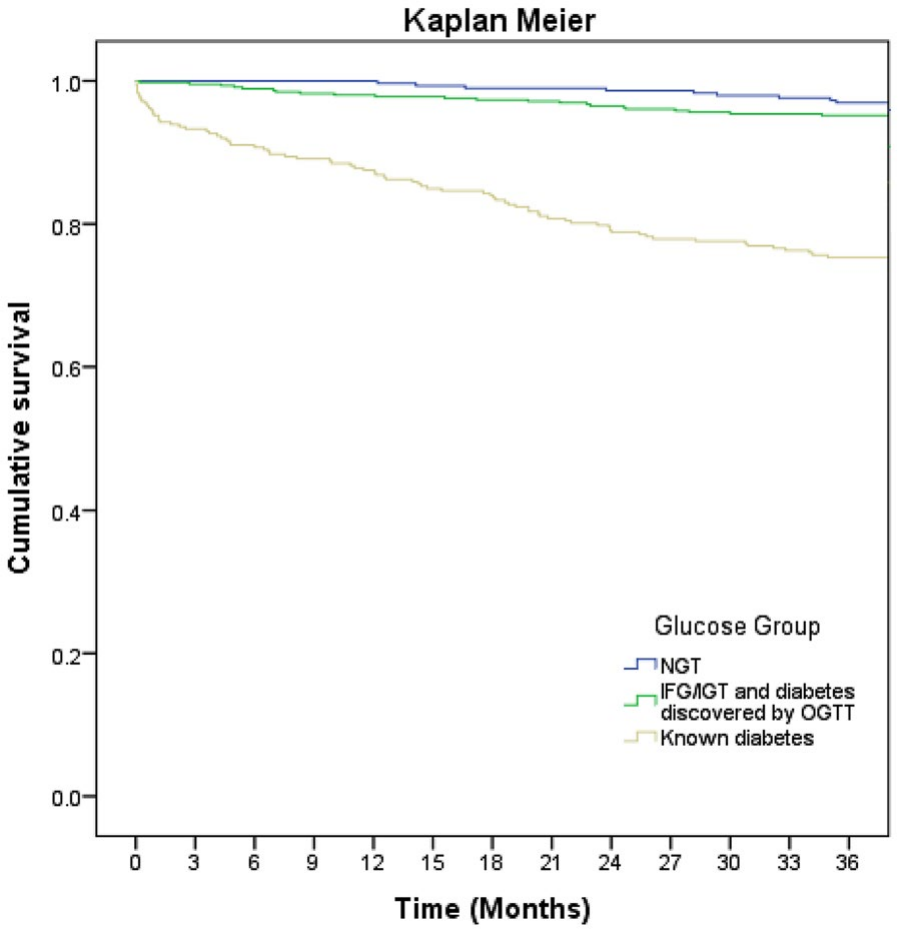
Kaplan–Meier curves showing cumulative mortality within 3 years. Kaplan–Meier curves showing cumulative death within 3 years. *Blue line* represents patients with NGT, *green line* patients with IFG + IGT and diabetes discovered by OGTT, and *beige line* patients with known diabetes at admission

Table 4 shows that there was significantly higher mortality within 30 days, 1 and 3 years in the group with known diabetes at admission compared to individuals with NGT and dysglycaemia (IFG, IGT and diabetes) discovered by OGTT (p < 0.001). The 3-year mortality among patients with type 1 diabetes was approximately 60 % (data not shown). Figure 1 shows the cumulative mortality within 3 years in relation to the various glucose tolerance groups, which include NGT, dysglycaemia discovered by OGTT, and patients with known diabetes. The cumulative mortality within 3 years was significantly higher in patients with known diabetes compared to the other two groups.

During a follow-up time of 4.0 (±0.8) years, 86 patients (28 %) with known diabetes had reinfarction as compared to 36 patients (12 %) with NGT and 79 patients (17 %) with dysglycaemia discovered by OGTT.

## Discussion

The results of the present study show that a majority of patients with ACS had dysglycaemia, in other words, known diabetes or disturbed glucose metabolism discovered by OGTT.

Patients with known diabetes had significantly higher short- and long-term mortality as compared to both patients with NGT and those with dysglycaemia discovered by OGTT; however, the latter group showed a non-significant trend towards higher long-term mortality as compared to the patients with NGT.

The baseline characteristics between patients with NGT and abnormal OGTT were not significantly different, while patients with known diabetes had significantly higher co-morbidity as seen with, for example, previous myocardial infarction, higher serum creatinine and BMI, which, in addition to higher age, can at least partly explain the poor outcome in these patients. Furthermore, the prevalence of systolic left ventricular (LV) dysfunction measured by echocardiography was significantly higher in patients with known diabetes. Echocardiographic parameters such as ejection fraction and myocardial strain reflecting LV systolic function, are associated with clinical outcome in patients with myocardial infarction [19, 20], and LV function is one the strongest predictors of outcome following PCI [21].

We found a high incidence of previously undiagnosed diabetes and/or IFG/IGT among patients with ACS. Notably, a substantial proportion of patients who died or had myocardial infarction during the follow-up were characterized as dysglycaemic, in other words, as having known diabetes at admission or disturbed glucose metabolism diagnosed by OGTT. Interestingly, the results indicate worsened prognosis, i.e. higher mortality and reinfarction for every stage that dysglycaemia progresses, that is, a trend towards higher mortality and reinfarction in patients with positive OGTT as compared to NGT, and significantly higher mortality and reinfarction in patients with known diabetes as compared to NGT. T2DM is preceded by disturbed glucose metabolism which at least at early stages may pass undiagnosed and untreated for several years. Disturbed glucose metabolism, also at early stages, seems to have a progressive adverse effect on the cardiovascular system reflected as poor clinical outcome, most significantly in patients with manifest diabetes. Early diagnosis and treatment of impaired glucose metabolism may slow down or even reverse the adverse effects on the cardiovascular system, while irreversible damages occur when impaired glucose metabolism progresses untreated to manifest diabetes.

Previous retrospective studies have investigated the prevalence of dysglycaemia and its influence on clinical outcome in patients with CAD [22–30]. However, the accuracy of the results of some of these studies is worthy of discussion either due to the methods that were used to diagnose diabetes, which included fasting blood glucose and HbA1c levels, or inclusion of patients with different diagnosis of CAD, such as, for example, stable vs ACS, associated with different short- and long-term prognosis. In the present study, we performed OGTT to investigate the glycaemic status of all patients with previously unknown DM. The European Diabetes Society and European guidelines on cardiology recommend the performance of OGTT in patients with CVD, especially in those with ACS. In patients with ACS, an abnormal OGTT seems to be a better prognostic marker than fasting blood glucose [31–34] or HbA1c alone [34].

Although the importance of hyperglycaemia as a predictor of survival in patients with ACS is well established, the association between dysglycaemia and mortality might differ across the spectrum of CAD [35]. In the present study, we included only patients with ACS, in other words, those with unstable angina and myocardial infarction.

In our study a substantial number of patients with known diabetes were treated conservatively; that is, significantly fewer patients underwent coronary angiography and PCI. The reason why a less invasive approach was chosen in the treatment of patients with known diabetes in this study is unclear. One reason could be that patients with known diabetes had already undergone coronary angiography during previous admissions since more patients in this group had previous MI, and perhaps based on previous examinations and/or decisions, a more conservative approach was chosen when the patient was readmitted with a new event. Another possible explanation concerns the risks of kidney dysfunction caused by the contrast given during coronary angiography and PCI, in other words, contrast-induced nephropathy (CIN), since the renal function was already significantly deteriorated in these patients as compared to patients in the two other groups. Higher age and existence of significant co-morbidities may also have played a role in choosing a more conservative approach. A non-invasive treatment strategy may have influenced the long-term prognosis in patients with known diabetes, as it has been demonstrated that an invasive strategy in the management of patients with ACS reduces long-term cardiovascular mortality and morbidity [36].

Although kidney dysfunction influences the likelihood of choosing an invasive treatment strategy in patients with ACS [37], previous studies have shown that an early invasive therapy is associated with better clinical outcome in patients with mild to moderate kidney dysfunction [38]. However, kidney function remains a significant prognostic marker of outcome following PCI [21]. The interrelationship between kidney dysfunction and treatment strategy as, for example, with invasive vs non-invasive approaches, in patients with diabetes and ACS has not been fully investigated.

Of note, a majority of patients with known diabetes who underwent PCI were treated with bare metal stents (BMS), and less than 20 % of the patients with dysglycaemia detected by OGTT received drug-eluting stents (DES), since they were treated as non-diabetic patients at the time of PCI. As demonstrated in our study, a significant number of patients with positive OGTT before discharge from the hospital were actually treated as non-diabetic patients at the time of admission and, more importantly, during coronary angiography and PCI, which may have influenced the treatment strategies such as stent choice, thus possibly contributing to worse clinical outcome as seen in these patients [21]. These findings highlight the necessity of investigating glycaemic status early in patients with ACS as, for example, before coronary angiography, since it has influence on therapeutic strategies such as PCI vs CABG, choice of stent, and more. However, since this study was performed, the use of DES has increased tremendously in our department as well as all over Sweden, and currently the majority of patients, with or without diabetes, receive DES.

Due to the lack of initial symptoms, the diabetes diagnosis may be delayed for several years, even after entering a diabetic state [39]. The duration and severity of the disease are of great importance for cardiovascular risk as well as other diabetic complications. Both improved metabolic and blood pressure control has been shown to decrease morbidity and mortality in patients with CAD. Glycated haemoglobin (HbA1c) is commonly used as a measure of metabolic control. There is a strong relationship between HbA1c levels and mortality in patients with CAD, which is independent of other risk factors [40, 41]. In patients with ACS, high HbA1c levels are associated with worse short-term outcome [22, 42], and elevated levels of HbA1c are associated with the progression and difficulty of CAD [43]. However, improved glycaemic control was not associated with a decreased incidence of macrovascular complications or mortality [44]. In the present study, there are no available data of HbA1c at the time of admission and/or during hospital stay, and no data regarding glycaemic and blood pressure control during follow-up.

Today there is no consensus among cardiologists how to treat patients with T2DM or IGT, and which glucose-lowering therapy is optimal after a coronary event. One large clinical trial found that treatment with insulin in patients with myocardial infarction was associated with an enhanced risk of recurrent nonfatal myocardial infarction or stroke, while treatment with metformin was more beneficial [45, 46].

Another study found that insulin treatment with or without oral glucose-lowering therapy was associated with higher long-term mortality in patients with DM undergoing coronary angiography [47]. T2DM patients are often treated with high insulin doses leading to weight gain and high blood pressure and, despite intensified insulin therapy, the treatment goals, which also include improved metabolic control and tight blood pressure control, are not reached.

A limitation of our study is when interpreting the results one should take into consideration that this is a retrospective single-center study analysing register data lacking information regarding long-term glycaemic control and incidence of CIN during the follow-up period which are important predictors of outcome in patients with ACS. Furthermore, the register data from SWEDEHEART and SCAAR does not provide information regarding changes of medical therapy including antidiabetic treatment during the follow-up period.

## Conclusions

The results of the present study show that undiagnosed dysglycaemia is common in patients with ACS. Both known diabetes and newly detected dysglycaemia showed increased risk for poor long-term clinical outcome, while short-term mortality was greater among patients with known diabetes. Routine OGTT in patients with ACS is useful for detecting undiagnosed dysglycaemia; however, new therapeutic approaches are needed to improve long-term prognosis in these patients.

## Abbreviations

ACS: acute coronary syndrome
AGEs: advanced glycation end products
BMI: body mass index
BMS: bare metal stent
CABG: coronary artery bypass graft
CAD: coronary artery disease
CIN: contrast induced nephropathy
CVD: cardiovascular disease
DES: drug-eluting stent
HbA1c: haemoglobin A1c
IFG: impaired fasting glucose
IGT: impaired glucose tolerance
LVEF: left ventricular ejection fraction
NGT: normal glucose tolerance
OGTT: oral glucose tolerance test
PCI: percutaneous coronary intervention
SCAAR: Swedish coronary angiography and angioplasty registry
SD: standard deviation
SWEDEHEART: Swedish web system for enhancement and development of evidence-based care in heart disease evaluated according to recommended therapies registry
T2DM: type 2 diabetes mellitus
